# Gradual weaning of 3-month-old calves from foster cows in dairy production

**DOI:** 10.3168/jdsc.2023-0470

**Published:** 2023-12-09

**Authors:** Margit Bak Jensen, Laura E. Webb, Mette Vaarst, Eddie A.M. Bokkers

**Affiliations:** 1 Department of Animal and Veterinary Sciences, Aarhus University, DK-8830 Tjele, Denmark; 2 Department of Animal Sciences, Animal Production Systems group, Wageningen University & Research, 6700AH, Wageningen, the Netherlands

## Abstract

When calves are reared by cows, separation can be stressful, and it is often advised to use gradual weaning to minimize this stress. In foster cow systems, one gradual weaning method is to remove the foster cows, one by one, from the cow-calf group. The response to one-by-one removal of foster cows of 32 calves housed in 5 groups was studied. Calves were fostered to a designated foster cow individually, in pairs, or in trios, at the age of 1 to 2 wk and then housed in groups of 4 to 6 foster cows and 6 to 11 calves until gradual weaning started at 3 mo of age. Calf behavior and aggression received by calves from cows were recorded using video during 18 h before any foster cow was removed, as well as 18 h before and 18 h after the designated foster cow was removed from the group. Before any foster cow was removed, the calves spent a similar amount of time sucking the designated foster cow and other cows (in total 43 ± 5 min/18 h). The total duration of sucking was similar before and after the designated foster was removed, as the duration of sucking other cows increased. The number of aggressive events received from other cows also increased. The results indicate that milk availability for the group of calves decreases as foster cows are gradually removed and that the older and heavier calves increasingly compete with the younger and lighter calves for the available milk. This weaning method may also compromise the welfare of the foster cows that are left to wean off an increasing number of calves, and this warrants further research.

## Highlights

• Before gradual weaning was initiated, the calves spent a similar amount of time sucking the designated foster cow and other cows.
• The total duration of sucking was similar before and after the designated foster was removed, as the duration of sucking other cows increased.
• The number of aggressive events received from other cows increased after the designated foster cow was removed.
• A longer total sucking duration was observed among beef-breed crosses compared with milk-breed calves.

## Graphical Abstract

**Figure:**
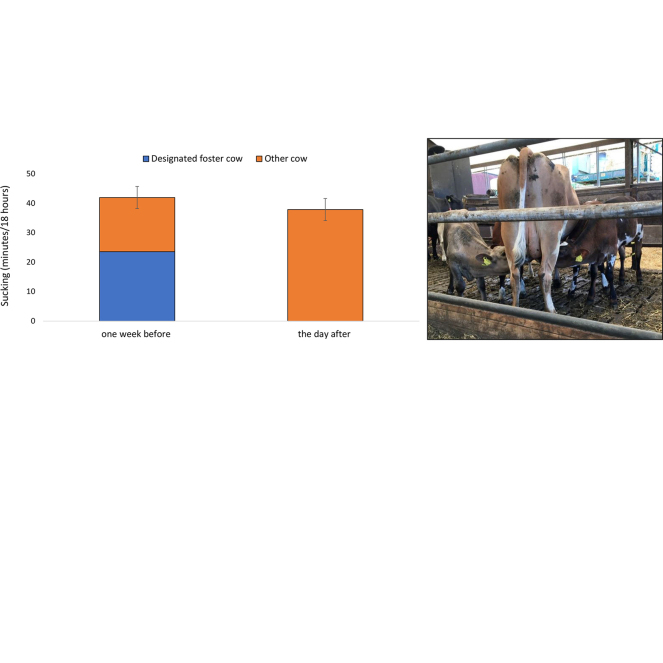
**Summary** This study investigated the effect of gradually weaning 3-month-old calves reared by foster cows. One week before weaning was initiated, the calves spent a similar amount of time sucking their designated foster cow and other cows. During gradual weaning, cows were removed, one by one, from the cow-calf group. Removing the designated foster cow reduced the calves' total time spent suckling, but calves received more aggression from other cows. Thus, calves intensified suckling of the foster cows left in the cow-calf group. This may compromise the welfare of both younger and lighter calves, as well as the cows left to wean off more and more calves.

Rearing dairy calves with their dam or a foster cow has several welfare benefits for the calf, including improved social skills, better learning ability and reduced occurrence of abnormal behavior (reviewed by Meagher et al., 2019). Rearing dairy calves with their dam comes at the cost of saleable milk (Barth, 2020), which is one of the reasons for interest in having a foster cow rear the calf instead of the dam (Bertelsen and Vaarst, 2023). A foster cow typically nurses one or more calves that are not her own (foster calves). The number of foster calves may vary depending on the foster cow's milk yield. The foster cows may be in any stage of lactation (Loberg and Lidfors, 2001) and may be cows with high cell counts. Foster cows are typically not machine milked (Sirovnik et al., 2020).

When calves are kept with their dam after birth, a maternal-filial bond is formed within the first 24 h, and the longer the two stay together within the first days to weeks, the stronger the response to separation will be in terms of vocalizations, pacing, and standing with the head out of pen (reviewed by Jensen, 2018). If the calf is separated from the dam within the first days or weeks of life and after that kept with a foster cow, the success of this cow-calf system depends on foster cows nursing the calves. The formation of a preferential bond between foster cow and calf facilitates nursing behavior. The relationship a foster cow may form with a calf can be categorized as adoption, acceptance, or rejection (Le Neindre 1982; Dunn et al., 1987). Adoption is characterized by the foster cow licking and nursing the calf in the inverse parallel position, which allows the cow to sniff and lick the calf's anogenital region while nursing, whereas acceptance is characterized by the calf being allowed to suck without the foster cow showing any aggressive behavior toward it (Dunn et al., 1987). Finally, rejection is characterized by the foster cow being aggressive toward the calf and not allowing it to suck (Loberg and Lidfors, 2001). Behavioral indicators of the level of a calf's bonding with a foster cow have not been reported. However, studies of dam-reared calves show that they lick their dam (Bertelsen and Jensen, 2023) and perform social play with their dam (Bailly-Caumette et al., 2023), whereas these behaviors were not directed toward other cows in these studies.

Špinka and Illmann (1992) found that young calves preferred to suck their own dam and made few attempts to suck other cows. When the authors removed a calf's dam, most calves preferred to suck one other cow. Among calves that were fostered to designated foster cows before being introduced to a group of foster cows and calves, Rosecrans and Hohenboken (1982) found that most calves sucked at least one other cow than their own foster cow, suggesting a less strong preferential bond between foster cow and calf than between dam and calf.

After the milk-feeding period, foster calves face a second separation: the separation from the foster cow and weaning off milk. It is generally recommended to gradually wean calves off milk, but because this is difficult in cow-calf systems, conducting the weaning off of milk and separation from the cow in 2 steps (using nose-flaps or fenceline weaning) to reduce weaning stress is advised in both beef (e.g., Price et al., 2003) and dairy production (Loberg et al., 2008; Johnsen et al., 2015; Wenker et al., 2022). However, when using foster cow systems, another approach to gradually wean calves off milk is to gradually reduce the number of cows nursing a group of foster calves. As mentioned previously, in foster cow systems, calves are often fostered to a dedicated foster cow. When gradually reducing the number of cows, the designated cows of the oldest calves may be removed first, with the intent to reduce milk intake of the oldest calves the most, and those of the youngest calves the least (as their designated foster is still present). When gradually weaning by removing foster cows successively, the question is whether those calves whose designated foster cow has been removed indeed suck less and thus experience the greater reduction in milk intake, or whether these calves will compensate by sucking other cows more and consequently impair the milk intake of the younger calves.

The aim of this study was to determine the effect of removing 3-mo-old calves' designated foster cow on calves' sucking behavior. The hypothesis was that foster calves would predominantly suck their designated foster cow. Further, we hypothesized that the duration of time calves spent sucking would be reduced after removal of their designated foster cow, and that calves would receive increased aggression from other cows, reflecting rejection of suckling.

The experiment was conducted on a commercial organic dairy farm in Jutland, Denmark, between June 2020 and April 2021, following the farm's standard management practice. A total of 43 calves (28 milk breed [all females; crossbred Danish Holstein, Danish Red, and Jersey] and 15 beef-crossbred calves [7 male and 8 females]) were included in the study. All calves were born in an individual straw-bedded calving pen (~18 m^2^) and stayed there with the dam for at least 24 h before being moved to a deep straw-bedded group pen for dams and their calves. Here dams were milked once daily in an automatic milking system. Dams and calves stayed in this group pen for 1 to 2 wk, whereafter calves were separated from their dam and moved to a straw-bedded pen together with an unfamiliar foster cow (milk breed: crossbred Danish Holstein, Danish Red, and Jersey), either alone (n = 5), with 1 other calf (n = 32; 16 pairs), or with 2 other calves (n = 6; 2 trios). Foster cows (all late-lactating higher-parity cows) were not milked. Each foster cow and her foster calves stayed in this pen (~18 m^2^) until nursing was well established. Thereafter, calves were moved with their foster cow into 1 of 2 group pens (170 m^2^). Three groups of 4, one group of 5, and one group of 6 foster cows and 6 to 11 calves were formed by adding foster cows and their calves to the group when calves were on average 14 (SD 6.4) days old. Calves (n = 43) and foster cows (n = 23) were housed in these groups until gradual weaning was initiated when calves were ~3 mo old. The age range was 28, 25, 20, 33, and 37 d for groups 1, 2, 3, 4, and 5, respectively). All calves of a particular group were removed before the first animals of the next group were moved to the pen.

The 2 group pens for foster cows and calves were deep straw-bedded pens with slatted concrete floors at the post and rail feed manger. A TMR (grass silage based) was provided twice daily for ad libitum availability, and water was available ad libitum from 1 self-filling water trough per pen. Calves had access to a separate feeding area (20 m^2^), where a standard calf concentrate, hay, and water were provided ad libitum. Above each group pen a camera (Hikvision, Hangzhou, China) with infrared light was fitted to enable behavioral recordings.

Calves were gradually weaned off milk by removing the foster cows one by one from the group pen. The first foster cow removed from the group was the cow fostered onto the oldest calf, calf pair, or calf trio in the group. Seven days after the first cow had been removed, the cow fostered onto the next oldest calves in the group was removed, and so on until only one foster cow was left in the group of calves; that is, the gradual weaning duration depended in the number of cows in the group (Figure 1). No calves were removed from the group until the observations were completed.

**Figure 1:**
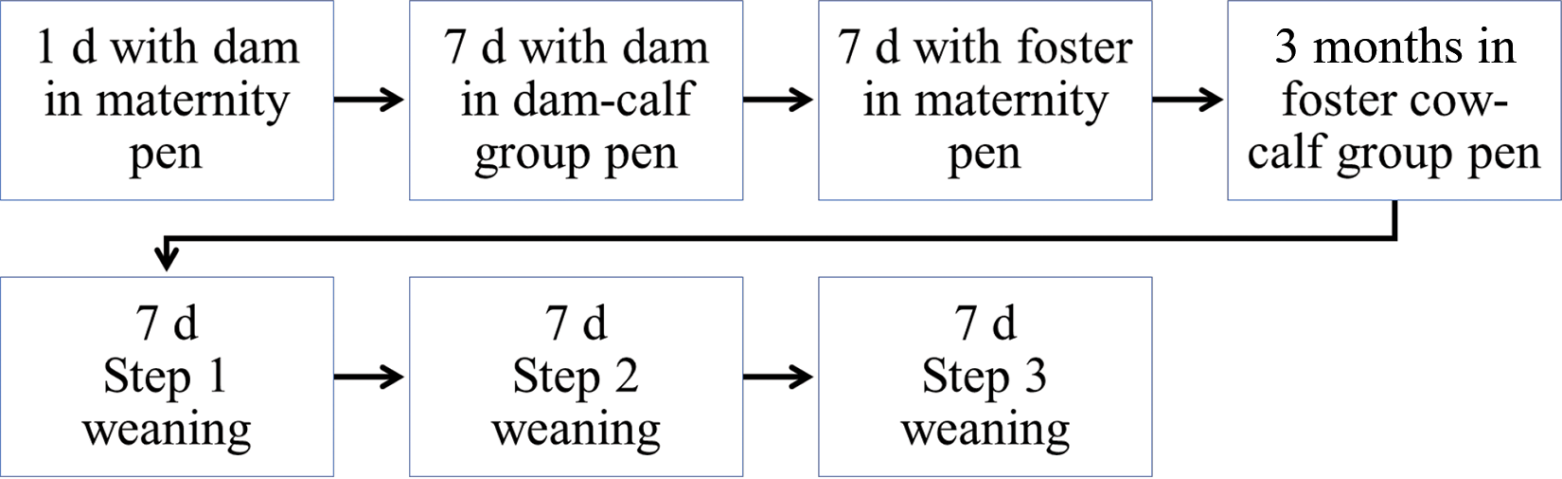
Timeline of the study. In step 1 of weaning, the cow fostered onto the oldest calf, calf pair, or calf trio in the group was removed. In step 2 of weaning, the cow fostered onto the next oldest calves in the group was removed, and so on until only 1 foster cow was left in the group of calves. The duration of weaning depended on the number of cows in the group (i.e., if there were 5 foster cows in the group, there were 4 steps in the gradual weaning).

For each of the 5 groups, the behavior of all calves (n = 43), including duration of sucking the designated foster cow and sucking another cow, as well as the frequency of being kicked by the designated foster cow and being kicked by another cow, was recorded continuously for 18 h (0400 to 2200 h) before any foster cow was removed from the pen (Table 1). At this moment calves were on average 91 (SD 15) days old. In addition, for 32 calves (1 calf per foster [n = 2], 2 calves per foster [n = 24; 12 pairs], or 3 calves per foster [n = 6; 2 trios]), duration of sucking the designated foster cow, sucking another cow, eating (concentrate, hay, or TMR), and lying, as well as the frequency of being kicked by another cow, were recorded continuously for 18 h (0400 to 2200 h) 6 d before and the day after the designated foster cow was removed from the group pen. When the designated foster was removed, the calves were on average 113 (SD 14) days old. The average BW of beef-breed crosses was 190 kg (SD 34.6), and the average BW of milk-breed calves was 167 kg (SD 25.2). All animals were marked for individual identification on the video recordings. All recordings were conducted using calf as the focal animal by the same observer, who was blind to the identity of the designated foster cow.

**Table 1:** Ethogram description of the recorded postures and behavior

Item	Description
Posture	
Lying	Calf is lying on sternum or side. Head may be rested or raised. The calf may be motionless or performing a behavior (e.g., self-grooming) while lying.
Upright	Calf's body supported by 3 to 4 legs, standing or walking.
Behavior while upright	
Sucking foster	The calf's head is under the foster cow's abdomen in the udder area. The behavior may be accompanied by butting directed toward the udder.
Sucking alien cow	The calf's head is under an alien cow's abdomen in the udder area. The behavior may be accompanied by butting directed toward the udder.
Eating	The calf is chewing, or feed (concentrate, hay, or TMR) is visible in the calf's mouth, or the calf's head is positioned in contact with feed or in close proximity of feed.
Other activity	Calf is performing any other behavior than listed above (e.g., grooming).
Aggression from foster	The calf is being kicked by the foster cow, or is forcefully being pushed by the foster cow placing its forehead to the body or head of the focal calf.
Aggression from alien cow	The calf is being kicked by an alien cow, or is forcefully being pushed by an alien cow placing its forehead to the body or head of the focal calf.

From the video recordings, the duration of time or frequency with which each calf performed each of the recorded behaviors was calculated for each 18-h period. In addition, the total duration of sucking (sucking designated foster cow plus sucking another cow) was calculated.

In the analysis of behavior before any foster cow was removed, the variables “duration of sucking designated foster cow” and “duration of sucking other cow” were analyzed using a paired *t*-test to test the hypothesis that the difference between the 2 is equal to zero: that is, that calves spent an equal amount of time sucking the designated foster and other cows. It was noted that 7 of 43 calves exclusively sucked the designated foster cow, and not any other cow, whereas the remaining calves sucked both the designated foster and at least one other cow. To test the effect of breed (which was confounded with sex and BW), the variables “sucking dam” and “total sucking duration” were analyzed using a linear mixed model (PROC MIXED, SAS V 9.4; SAS Institute Inc., Cary, NC), including breed (milk or beef-breed-cross) as fixed effect and calf age as a covariate. Group and foster subgroup were included as random effects.

In the analysis of behavior 6 d before and the day after removal of the designated foster cow, the variables “total sucking duration,” “sucking another foster cow,” “eating duration,” and “lying duration” were analyzed using a linear mixed model, whereas the number of aggressions received from other cows was analyzed by mixed logistic regression (using GLIMMIX, with Poisson distribution and log link). The models included as fixed effects day (before, after; i.e., 6 d before and the day after removal), breed (milk or beef-breed-cross), and day × breed interaction, and calf age was included as a covariate. Group and foster subgroup were included as random effects.

In all analyses, denominator degrees of freedom were calculated using the Kenward-Roger approximation. Significance was declared when *P* < 0.05, and tendencies were considered when 0.05 ≤ *P* < 0.10.

Before any foster was taken out of the group, we could not reject the null hypothesis of no difference between sucking a designated foster and sucking another cow duration (*t*-test _[df = 42]_ = 0.71; *P* = 0.48). The mean duration of sucking the foster cow was 23.1 (SD 17.4) min/18 h, and the mean duration of sucking another cow was 19.7 (SD 17.9) min/18 h. We detected no significant effect of either breed or age on any of the previously mentioned variables. A total of 65% of calves received aggression from at least 1 other cow, and 16% of calves received aggression from the designated foster cow during the observation before any foster was taken out of the group (these 2 variables are not formally analyzed).

As illustrated in Figure 2, no difference in the total duration of sucking was found between 6 d before and the day after removal of the designated foster cow (*F*_1,28_ = 1.15; *P* = 0.29), because the duration of sucking another cow increased from before the designated foster cow was removed to the day after (*F*_1,28_ = 21.5; *P* < 0.001). The number of aggressions from other cows toward calves from which the designated foster was removed also increased from 6 d before to the day after the designated foster cow was removed (1.45 ± 0.39 vs. 2.17 ± 0.54; *F*_1,29.7_ = 5.16; *P* = 0.03). Across days, a longer total sucking duration was observed among beef-breed crosses compared with milk-breed calves (45.9 ± 4.74 vs. 32.5 ± 4.07 min/18 h; *F*_1,25.9_ = 5.59; *P* = 0.026). Beef-breed crosses also spent longer sucking another cow than milk-breed calves across days (33.2 ± 4.18 vs. 21.5 ± 3.62 min/18 h; *F*_1,25.2_ = 5.77; *P* = 0.024). No effects of either day or breed were found on the duration of lying 584 (SD 80.8) min/18 h, nor the duration of eating 49 (SD 22.3) min/18 h.

**Figure 2:**
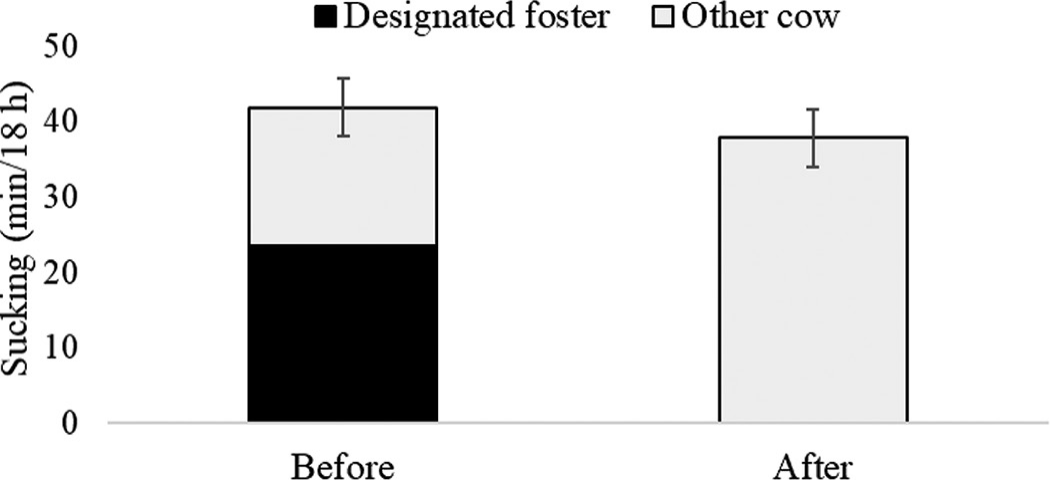
Duration of sucking the designated foster cow (black part of bar) and another foster cow (gray bars) by calves 6 d before the designated foster was removed and on the day after the designated foster was removed. Bars represent LSM, and error bars indicate SE.

Our first hypothesis was that foster calves would predominantly suck their designated foster cow. Before any foster cow was taken out of the group, and when calves were on average 3 mo old, on average half of the time spent sucking was directed to another cow. In comparison, other studies have shown that among dam-reared calves only a few calves suck cows other than their dam (e.g., Edwards, 1983; Bertelsen and Jensen, 2023). The maternal-filial bond is defined a mutual preferential relationship (Newberry and Swanson, 2008), whereas the relationship between a foster cow and a calf has been categorized as adoption (i.e., preferential relationship similar to a maternal bond) or merely acceptance (Dunn et al., 1987). Bertelsen and Jensen (2023) described how dams pushed their calves away from other cows when the calves tried to suck these other cows, and in the present study the high level of sucking another foster cow than the designated foster cow may be due to foster cows not interacting with their foster calves this way because they have merely accepted rather than adopted them. Whether the calves were accepted or adopted was not investigated in the present study. In addition, a foster cow that has not adopted but has merely accepted her designated foster calf or calves may be less likely to reject other calves, resulting in a greater likelihood that she nurses other calves. However, even if the foster cow has adopted her foster calves, the fact that a foster cow typically has more than one calf fostered onto her may make it difficult for her to reject a non-foster calf when one or more foster calves are already sucking. That a dam nurses a calf in the inverse parallel position has been proposed as an indicator of whether or not a maternal bond is formed (Waltl et al., 1995), and we encourage future studies to determine the nature of the relationship formed between foster cow and calf.

The high level of sucking cows other than the designated foster cow is likely driven by the calf. If the calf's relationship to a foster cow is less strong than the relationship that is formed with the dam soon after birth, then the calf may be more likely to suck other cows than the designated foster. In this case calves may choose to suck the cows with the highest yield or the cows that do not reject them. It may be argued that calves are more opportunistic than the dam, and sucking other cows, as well as the dam, may be a good strategy should the dam yield little milk. However, the maternal behavior of the dam during the hours following calving likely guides the calf. Illustrating the role of the calf's experience in sucking, calves that had been left undisturbed with their dam in an individual pen for the first 3 d after birth were more insistent in attempting to suck and more successful in sucking a late-lactating foster cow compared with calves that had been fed dam's milk via a teat bucket during the first 3 d (Vaarst et al., 2001).

The high level of sucking other cows than the designated foster cow in the present study may be due to the use of late-lactating cows. Maternal responsiveness is at its highest around the time of calving, but calves may be successfully fostered onto cows after that, such as 3 to 5 d after a cow has given birth to her own calf (Kent, 1984). Accordingly, Loberg and Lidfors (2001) showed that more cows had to be tethered to accept the calves' sucking when introduced to their foster calves at 178 d in lactation compared with cows that were introduced to the calves on the day of calving or 4 d after calving, whereas cows introduced to their foster calves 26 d after calving were intermediate. Thus, using late-lactating cows as foster cows increases the risk of foster cows merely accepting or even rejecting the foster calves, rather than adopting them. The variation in the percentage of sucking directed to cows other than the designated foster cow showed large variation, which may suggest that many calves in the present study were accepted rather that adopted. This may be because not all cows are appropriate as foster cows, but, even if a foster cow has adopted one or more foster calves, she may have been merely accepting or even rejecting other foster calves.

Our second hypothesis was that the duration of time calves spent sucking would be reduced once their designated foster cow was removed. However, when the designated foster cow was removed, calves responded by increasing the level of sucking other cows, resulting in an unchanged total duration of sucking from 6 d before to the day after their designated foster cow was removed. Noteworthy, at this stage, beef-breed crosses, which were heavier than milk-breed calves, spent longer sucking other cows, and no such effect was seen before any foster was removed from the group. No measure of milk intake was collected, and thus we cannot say whether the designated foster calves, milk-breed calves, or all calves, ingested less milk when a foster cow was taken out of the group; but because a cow's yield is unlikely to have increased, on average calves will have had a reduced milk intake. The gradual weaning method of removing the foster cows of the oldest calves first was intended to defend the intake of the youngest calves. Instead a considerable risk exists that the youngest calves could suck less milk due to the competition for udder access and milk of older calves now lacking a designated foster cow. This is supported by the finding that the heavier beef-breed crosses spent longer sucking in the present study. Had calves been selectively sucking their designated foster, the duration of time calves spent sucking would likely have been reduced once their designated foster cow was removed. Stronger bonding may have been possible if newly calved cows had been used as foster cows, or if cows and calves had been left for longer in their “bonding pen.” In addition, the surprising result that calves only spent approximately half of the time sucking on the designated foster cow resulted in that the calves maintained their sucking time when their designated foster was removed. Another aspect is the high individual variation in time sucking other cows; some calves more selectively sucked their designated foster than other calves. This could be because they were better bonded to their foster cow or because their foster had a higher milk yield.

Calves experienced an increase in received aggression from other cows on the day after their designated foster was removed from the group, compared with 6 d before. This likely indicates that they received more nursing rejections. Because we only observed the calves 6 d before and the day after their designated foster was removed (and not all calves in the group on that particular day), we cannot say whether only the observed calves, or all calves, received more aggression and rejections when fewer foster cows were left to nurse all calves in the group. It should also be noted that we recorded behavior the day after removal and did not follow the calves on the following days and weeks. The situation might change with time, and future studies are encouraged to investigate this aspect.

Total sucking time was unchanged, but we do not know whether calves were sucking efficiently and maintained milk intake after the removal of their designated foster. Whether the cows remaining in the group are able to regulate nursing by increasing rejections of certain, or all, calves, and the welfare consequences for the remaining cows of this gradual weaning method, warrant further study.

In conclusion, in groups of late-lactating foster cows and calves, where 1 to 3 calves had been fostered onto a designated foster cow, calves sucked on average 54% of the time from their designated foster cow, whereas the remaining sucking time was spent sucking other cows at the age of 3 mo. When a designated foster cow was removed, her calves did not show a reduction in sucking time, because they spent more time than before sucking other cows, but they also received more aggression from other cows, and therefore this management cannot be recommended. The heavier beef-breed crosses spent longer sucking other cows than the lighter milk-breed calves, and because the total available milk for all calves in the group must have been less, younger and lighter calves likely experienced the highest reduction in milk intake.
